# Financial Fraud Victimization Among Young Adults in Taiwan: Roles of Executive Functioning, Personality Traits, and the Self‐Reported Attention‐Deficit/Hyperactivity Disorder Diagnosis

**DOI:** 10.1002/brb3.71780

**Published:** 2026-09-23

**Authors:** Yi‐Chun Yeh, Pai‐Cheng Lin, Chung‐Ying Lin, Yu‐Ping Chang, Peng‐Wei Wang, Cheng‐Fang Yen

**Affiliations:** ^1^ Department of Psychiatry Kaohsiung Medical University Hospital Kaohsiung Taiwan; ^2^ Department of Psychiatry, School of Medicine, College of Medicine Kaohsiung Medical University Kaohsiung Taiwan; ^3^ Department of Psychiatry Kaohsiung Medical University Gangshan Hospital Kaohsiung Taiwan; ^4^ Institute of Allied Health Sciences, College of Medicine National Cheng Kung University Tainan Taiwan; ^5^ Biostatistics Consulting Center, National Cheng Kung University Hospital, College of Medicine National Cheng Kung University Tainan Taiwan; ^6^ Department of Public Health, College of Medicine National Cheng Kung University Tainan Taiwan; ^7^ Department of Occupational Therapy, College of Medicine National Cheng Kung University Tainan Taiwan; ^8^ School of Nursing The State University of New York, University at Buffalo Buffalo New York USA; ^9^ College of Professional Studies National Pingtung University of Science and Technology Pingtung Taiwan

**Keywords:** attention‐deficit/hyperactivity disorder, executive function, Big Five Inventory, financial fraud

## Abstract

**Background:**

Financial fraud victimization can lead to financial devastation and is associated with mental health problems. The current study examined differences in executive function, personality traits, and the rate of a self‐reported attention‐deficit/hyperactivity disorder (ADHD) diagnosis across varying experiences of financial fraud victimization among young adults in Taiwan.

**Methods:**

A total of 1764 young adults participated in this study. The participants reported their experiences with 11 types of financial fraud over the past 5 years. The participants were categorized into the following three groups on the basis of their exposure to financial fraud and whether they lost money: no exposure to fraud, exposure without monetary loss, and exposure with monetary loss. The executive function, personality traits, and the rate of a self‐reported ADHD diagnosis were compared among the three groups using multivariate analysis of covariance.

**Results:**

Of the participants, 47.6% had been scammed out of money, 26.9% had experienced fraud without monetary loss, and 25.5% had had no exposure to financial fraud. The participants who had been scammed were more likely to self‐report having an ADHD diagnosis and reported worse working memory and inhibition than did the participants in the other groups (all *p* < 0.001). The participants exposed to fraud without monetary loss reported worse working memory than did those with no exposure (*p* < 0.001). The participants who had no exposure to fraud scored higher in terms of extraversion (*p* = 0.010), agreeableness (*p* < 0.001), conscientiousness (*p* < 0.001), and openness (*p* = 0.023) than did those who had been scammed, whereas the participants who had been scammed scored higher in terms of neuroticism than did those with no exposure (*p* = 0.009).

**Conclusion:**

Differences in executive functioning, personality traits, and the rate of a self‐reported ADHD diagnosis were observed among the young adults with varying experiences of financial fraud victimization.

## Graphical abstract

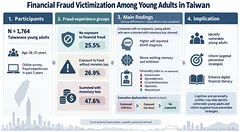

## 1 Introduction

The Centers for Disease Control and Prevention defines fraud as “an intentional distortion of truth initiated to convince another to part with something of value or to surrender a legal right” (Hall et al. 2016). Fraud is typically committed to achieve personal gain by causing harm to another party (Hall et al. 2016). The occurrence of financial fraud victimization is increasing globally, as indicated in numerous reports from the United States (Burnes et al. 2017), China (Fan and Yu 2021), Japan (Ueno et al. 2021), Hong Kong (J. C. M. Li et al. 2022), and various developing countries (Bar Lev et al. 2022). Studies have indicated that financial fraud victimization not only causes financial devastation (Brenner et al. 2020; DeLiema et al. 2017; Knupfer et al. 2021) but also leads to severe stress and anxiety (Button et al. 2014), psychotraumatic symptoms (Hamby et al. 2021), low subjective well‐being (Hamby et al. 2021), decreased health‐related quality of life (Hamby et al. 2021), and feelings of self‐blame, shame, and embarrassment (Button et al. 2014; Cross 2015). However, although the effects of financial fraud are far‐reaching and pervasive, researchers still know little about why individuals become victims of fraud (Hanoch and Wood 2021).

Most studies on financial fraud have focused on older adults (Burnes et al. 2017; Fan and Yu 2021; J. C. M. Li et al. 2022; Ueno et al. 2021). Older individuals often experience cognitive decline (Han et al. 2015a) and possess outdated financial knowledge (Han et al. 2015b), which increases their vulnerability to financial fraud (Boyle et al. 2012). However, financial fraud victimization is not limited to the older population; other age groups, including young adults, are also at high risk (DeLiema et al. 2024). The risk of financial fraud among young adults should be considered for several reasons. First, many young adults are newly managing their finances and may not have developed risk management skills. Second, they are more likely than older adults to make purchases online, which is a key channel through which financial fraud occurs (Ueno et al. 2021). Identifying the factors associated with financial fraud among young adults is crucial in developing effective prevention strategies.

Studies on financial fraud have generally categorized participants as either fraud victims or nonvictims and compared the associated risk factors between these groups (Burnes et al. 2017; Fan and Yu 2021; J. C. M. Li et al. 2022; Ueno et al. 2021). However, no study to date has compared risk factors among individuals who had no exposure to fraud, those who were exposed but did not lose money, and those who were scammed out of money. Compared with individuals who were scammed out of money, those who were exposed but did not lose money may have a keen ability to spot scams, which prevent them from being scammed. Nevertheless, compared with individuals who had no exposure to fraud, those who were exposed to fraud may not be careful enough and may leak their personal data to fraudsters. Examining risk factors on the basis of individuals’ varying experiences with financial fraud exposure can help explain why some people are targeted and why some ultimately lose money.

Cognitive decline plays a crucial role in the vulnerability to financial fraud among older adults (DeLiema et al. 2017; Judges et al. 2017). Studies have determined that older fraud victims have lower levels of cognitive abilities, including with respect to language, memory, attention/concentration, processing speed, visuospatial ability, and executive functioning, than do nonvictims (DeLiema et al. 2017; Ebner et al. 2020; Han et al. 2016; Judges et al. 2017). Working memory and inhibitory control are two core components of executive function that help individuals plan, organize, and manage tasks to achieve goals (Diamond 2013). Working memory refers to the brain system responsible for the temporary storage and manipulation of information required for complex cognitive tasks (Baddeley 1992). Inhibitory control refers to the ability to regulate one's efforts to override strong internal impulses or external temptations (Diamond 2013). Studies have observed that working memory and inhibitory control are strongly associated with the ability to detect deception in older individuals (Calso et al. 2020; Judges et al. 2017). By contrast, cyber‐fraud victims tend to have a higher level of disinhibition, exhibiting traits such as urgency and sensation seeking (Whitty 2019). Whether working memory and inhibitory control are associated with financial fraud victimization in young adults remains unclear and warrants further investigation.

Several studies have examined the association of personality traits with vulnerability to financial fraud among older adults. Judges et al. (2017) determined that the personality trait of conscientiousness, which reflects how organized, responsible, and detail‐oriented a person is, was associated with a lower risk of fraud victimization in older adults. Xing et al. (2020) reported that agreeableness, that is, being cooperative, kind, polite, and friendly, was also associated with a lower risk of fraud in older adults. Whether certain personality traits are associated with financial fraud, victimization in younger adults remains to be investigated.

Young adults with attention‐deficit/hyperactivity disorder (ADHD) experience cognitive dysfunction in attention and decision‐making (Kooij et al. 2010). They exhibit increased tendencies toward risky decision‐making, impaired reinforcement learning, and a preference for immediate over delayed rewards (Mowinckel et al. 2015). Further, individuals diagnosed with ADHD exhibited difficulties in working memory and response inhibition (Townes et al. 2023), which may increase the risk of fraud victimization among individuals with ADHD. However, whether young adults with a self‐reported ADHD diagnosis have a higher risk of financial fraud victimization than do those without a self‐reported ADHD diagnosis remains to be examined.

According to the 2024 Asia Scam Report (Global Anti‐Fraud Alliance 2024), as many as 66% of people in Taiwan encounter at least one attempt at fraud every month. The average monthly financial losses suffered by Taiwanese citizens as a result of financial fraud in 2025 amounted to nearly 8 billion New Taiwan dollars (National Police Agency, Ministry of the Interior 2025). The main reasons for the rampant prevalence of fraud in Taiwan lie in the severe leakage of personal data, the lack of source‐level management of social media and messaging apps, and the highly globalized nature of the fraud industry. Coupled with the extremely high internet usage rate among the Taiwanese public and the proliferation of social media advertising, these factors enable fraud syndicates to deliver phishing messages with pinpoint accuracy, leaving the public unable to guard against them (Academy for the Judiciary, Ministry of Justice 2023).

The present study investigated the types of financial fraud exposure and the channels through which scams occurred among young adults in Taiwan. In addition, this study examined differences in executive function, personality traits, and the rate of a self‐reported ADHD diagnosis among the following three groups, categorized on the basis of their experiences with financial frauds: no exposure to fraud, exposure without monetary loss, and exposure with monetary loss. We hypothesized that individuals who were scammed out of money had the worst executive function and highest neuroticism, followed by those with exposure but without monetary loss and those with no exposure. We also hypothesized that individuals who were scammed out of money had the lowest extraversion, agreeableness, conscientiousness, and openness to experiences, followed by those with exposure but without monetary loss and those with no exposure.

## 2 Methods

### 2.1 Participants and Procedures

We recruited Taiwanese young adults aged 18–25 years through online advertisements posted on Dcard. Dcard is the most widely used social media platform among young adults in Taiwan and has up to 15 million unique visitors per month, with a total of over 10 million registered accounts. Active users are mainly aged between 18 and 35, of whom nearly 40% are already in full‐time employment. Participant recruitment was conducted between March and June 2025. The advertisement for the study included detailed information regarding the study's objectives, instructions for completing the online questionnaire, and assurances of data confidentiality and participant privacy. Individuals interested in participating were able to access the questionnaire by clicking an “Agree to Participate” button, after which they proceeded to provide their responses. Those who did not wish to participate could opt out by selecting a “Not Willing to Participate” button or by simply ignoring the advertisement. In total, 1764 young adults participated in the study.

### 2.2 Ethics Statement

The study protocol was approved by the Institutional Review Board of Kaohsiung Medical University Hospital (KMUHIRB‐E(I)‐20240422). This was a questionnaire‐based study, and no experiments involving human participants or human tissue samples were conducted. This study was conducted in accordance with the ethical principles outlined in the Declaration of Helsinki and followed the guidelines for the conduct, reporting, editing, and publication of scholarly work in medical journals.

### 2.3 Measures

#### 2.3.1 Financial Fraud Experiences

This study surveyed participants on the types of financial fraud they had experienced in the past 5 years. The survey on experiences of financial fraud over the past 5 years was conducted for the following reasons. First, the proportion of Taiwanese people who have fallen victim to financial fraud has risen significantly since 2021. Second, as the study focused on young adults, a 5‐year survey period closely aligned with the participants’ financial management experiences. To identify common types of financial fraud in Taiwan, we posted an open‐ended question on social media asking Taiwanese individuals to share their personal experiences with fraud or incidents that they had heard of. There were a total of 436 comments regarding the types of experiences of financial fraud. Three researchers each carefully read through these comments and carried out an initial classification; they then discussed the findings, comparing and consolidating their classifications. This study also referred to the fraud categorization frameworks proposed by Ueno et al. (2021) and Kemp and Erades Pérez (2023). Finally, we identified the following 11 types of financial fraud as being common in Taiwan: “It's Me” fraud, kidnapping fraud, refund fraud, bank or cell phone account fraud, billing fraud, shopping fraud, financial investment fraud, lottery fraud, job‐hunting fraud, romance fraud, and fortune‐telling fraud. Detailed descriptions of the Financial Fraud Experiences Questionnaire for each fraud type are provided in Table 1. The participants were asked to respond to each item with “no” or “yes” to indicate whether they had experienced a given type of fraud. If participants selected “yes,” they were asked a follow‐up question about whether they had been scammed out of money, with response options for this question also being “no” or “yes.” A pilot study examining the financial fraud experiences of 1822 individuals in Taiwan supported the one‐factor structure of the Financial Fraud Experiences Questionnaire. The first subsample was used for exploratory factor analysis (EFA; *n* = 893) and the second for confirmatory factor analysis (CFA; *n* = 929). In the EFA, Kaiser's rule (i.e., eigenvalue > 1) was adopted to decide the number of extracted factors, and the extraction method was principal axis factoring using polychoric correlation. The first eigenvalue extracted from the EFA subsample was 3.74 and the second eigenvalue was 0.71. Moreover, all EFA factor loadings were larger than 0.4 (please see Table S1 for details). Therefore, the one‐factor structure was used for the CFA. Fit statistics of CFA were all acceptable, except for the significant *χ* ^2^ test (please see Table S1 for details). Therefore, findings from EFA and CFA support the one‐factor structure of the Financial Fraud Experiences Questionnaire. Furthermore, both forms of internal consistency for the Financial Fraud Experiences Questionnaire were acceptable: *α* = 0.847 and *ω* = 0.878. In this study's statistical analysis, the participants were divided into three groups: those with no exposure to fraud (Group A), those exposed to fraud but not scammed out of money (Group B), and those who had been scammed out of money (Group C).

**TABLE 1:** Financial fraud types and descriptions.

Types	Description
“It's Me” fraud	Scammers pose as a relative or other family member and request that you transfer money to a designated account.
Kidnapping fraud	Scammers claim to have kidnapped a family member and demand a ransom.
Refund fraud	Scammers impersonate government officials processing refunds for overpaid medical, tax, or insurance expenses and request your PIN or a money transfer to a designated account.
Bank or cell phone fraud	Scammers pose as officials of banks or cell phone companies and ask for your PIN or a monetary transfer to a designated account.
Billing fraud	Scammers inform you that you have yet to pay a fake bill.
Shopping fraud	You pay for an item but either do not receive it or receive something you did not order.
Financial investment fraud	You are cheated when investing in stocks, funds, virtual money, columbarium, or similar schemes.
Lottery fraud	Scammers inform you that you have won a prize and ask you to pay a fee to redeem the prize.
Job hunting fraud	You are asked to pay for goods and services when you are looking for a job.
Romance fraud	You are scammed out of money by someone pretending to be a romantic partner.
Fortune‐telling fraud	Scammers ask you to pay money to know or change your future.

The participants were also asked to indicate the channels through which the financial fraud occurred. The options included cell phone or telephone, email, mobile telecommunication apps (e.g., Line and Facebook Messenger), social media platforms (e.g., Facebook, Instagram, TikTok, YouTube, X, and Threads), online advertisements, and non‐web‐based methods such as multilevel marketing presentations, referrals from friends or family, and invitations from strangers (Kemp and Erades Pérez 2023).

#### 2.3.2 Personality Traits

This study used the shortened version of the Big Five Inventory (BFI) to assess five dimensions of personality, namely, extraversion (three items), agreeableness (three items), conscientiousness (three items), neuroticism (three items), and openness to experiences (three items; John et al. 1991; R.‐H. Li and Chung 2020). Each item is rated on a 5‐point scale with endpoints ranging from 1 (*strongly disagree*) to 5 (*strongly agree*), with higher subscale scores indicating stronger tendencies toward the corresponding personality traits. The shortened BFI demonstrated acceptable reliability and validity in the Taiwanese population (R.‐H. Li and Chung 2020). In the present study, the Cronbach's *α* values for the five subscales ranged from 0.79 to 0.87.

#### 2.3.3 Executive Functioning

This study used the Adult Executive Functioning Inventory (ADEXI) to assess two dimensions of executive function, namely, working memory (nine items; e.g., “I have difficulty remembering lengthy instructions”) and inhibition (five items; e.g., “I have a tendency to do things without first thinking about what could happen”) (Holst and Thorell 2018). Each item is rated on a 4‐point scale with endpoints ranging from 0 (*definitely not true*) to 3 (*definitely true*), with higher subscale scores indicating worse working memory and inhibition. The ADEXI demonstrated acceptable reliability and validity (Holst and Thorell 2018). In the present study, Cronbach's *α* was 0.91 for the working memory subscale and 0.71 for the inhibition subscale.

#### 2.3.4 Self‐Reported ADHD

We assessed the self‐reported ADHD diagnosis by using a single question: “Since you were a child, have you ever received a diagnosis of or been suspected of having ADHD?” Participants responded with either “yes” or “no.”

#### 2.3.5 Sociodemographic Characteristics

This study collected data on the participants’ sex (men, women, and nonbinary), age, education level (high school or below vs. college or above), and sexual orientation (heterosexual vs. gay, lesbian, bisexual, or other).

### 2.4 Statistical Analysis

Descriptive statistics were calculated to summarize the participants’ demographic characteristics, experiences of financial fraud victimization, a self‐reported ADHD diagnosis, and scores on the ADEXI and BFI. The chi‐square test and analysis of variance (ANOVA) were used to compare the demographic characteristics and presence of a self‐reported ADHD diagnosis among the three groups, namely, those who had no exposure to financial fraud (Group A), those who had exposure to financial fraud but did not lose any money (Group B), and those who had exposure to financial fraud and lost money (Group C). Multivariate analysis of covariance (MANCOVA) was used to compare the executive function and personality traits among the three groups. This study recruited young adults through advertisements on Dcard. Each internet protocol address could only complete the survey once. Through this approach, this study ensured, to the greatest extent possible, that each data point was separate and random and that one participant did not influence another. Independence of observations was addressed through the study design by permitting only one survey submission per IP address.

Univariate normality was assessed using skewness and kurtosis values and graphical inspection of the distributions (Kim 2013). Multivariate normality was evaluated using Mahalanobis distance and corresponding chi‐square plots (Wan Nor 2015). Linearity among the dependent variables was assessed by visual inspection of scatterplot matrices within the financial fraud victimization groups (Tabachnick and Fidell 2018). Multicollinearity was evaluated by examining Pearson correlations among the dependent variables (Tabachnick and Fidell 2018). No substantial nonlinear relationships were observed, and the correlations among the dependent variables did not indicate problematic multicollinearity.

Homogeneity of error variances for individual dependent variables was assessed using Levene's test. Levene's tests were significant except for working memory and conscientiousness, indicating that the assumption of homogeneity of variance was not fully satisfied. Equality of covariance matrices was assessed using Box's *M* test. Box's *M* tests were significant for both the executive functioning model (Box's *M* = 36.282, *p* = 0.002) and the personality traits model (Box's *M* = 268.381, *p* < 0.001), also indicating heterogeneity of covariance matrices (Tabachnick and Fidell 2018). Given the observed heterogeneity of variances and covariance matrices, Pillai's trace was used as the primary multivariate test statistic because of its relative robustness to violations of these assumptions (Tabachnick and Fidell 2018). Then the effects of groups on each domain of executive function and personality traits were examined. Post hoc pairwise comparisons were done if the between‐group effect was significant. To account for multiple comparisons, the significance of all ANOVAs and MANCOVAs was adjusted using the Sidak correction. A *p* value < 0.05 was considered significant. Partial *η* ^2^ and standardized differences (Cohen's *d*) for key contrasts were the indicators for effect sizes. The values of partial *η* ^2^ 0.01, 0.06, and 0.14 represent small, medium, and large effect sizes, respectively. Cohen's *d* values of 0.2, 0.5, and 0.8 represent small, medium, and large effect sizes, respectively. In addition, sensitivity analyses using univariate general linear models with HC3 heteroskedasticity‐consistent standard errors were conducted for each dependent variable, with financial fraud victimization group and education level entered as categorical factors (Tabachnick and Fidell 2018). The results were reported in Table S2.

## 3 Results

Table 2 presents data regarding the participants’ sociodemographic characteristics, self‐reported ADHD diagnosis, executive functioning, and personality traits. Table 2 also presents the mean (SD) scores for the levels of working memory and inhibition, as measured using the ADEXI, as well as the five personality traits, as assessed using the BFI.

**TABLE 2:** Participant sociodemographic characteristics, self‐reported attention‐deficit/hyperactivity disorder (ADHD), executive functioning, and personality traits (*N* = 1764).

	*n* (%) or mean (SD)	Range
Gender, *n* (%)		
Women	986 (55.9)	
Men	778 (44.1)	
Age (years), mean (SD)	22.3 (1.8)	18–25
Sexual orientation, *n* (%)		
Heterosexual	1448 (82.1)	
Gay, lesbian, bisexual, or others	316 (17.9)	
Education level, *n* (%)		
High school or below	236 (13.4)	
College or above	1528 (86.6)	
Having self‐reported ADHD		
No	1703 (96.5)	
Yes	61 (3.5)	
Working memory on the ADEXI	23.0 (6.5)	9–45
Inhibition on the ADEXI	13.7 (3.4)	5–25
Extraversion on the shortened BFI	9.9 (2.5)	3–15
Agreeableness on the shortened BFI	10.2 (2.4)	3–15
Conscientiousness on the shortened BFI	11.3 (2.1)	3–15
Neuroticism on the shortened BFI	8.9 (2.7)	3–15
Openness to experience on the shortened BFI	9.5 (2.4)	3–15

Abbreviations: ADEXI, Adult Executive Functioning Inventory; BFI, Big Five Inventory.

Table 3 presents the types of financial fraud exposure reported by participants. The three most commonly experienced types of financial fraud were bank or cell phone account fraud (35.8%), billing fraud (33.8%), and refund fraud (31.9%). Shopping fraud was the most common type in which participants were scammed out of money (15.9%). The most common channel through which financial fraud occurred was through cell phone or telephone (34.8%), followed by mobile telecommunications (31.5%), social media (23.0%), email (14.9%), online advertisements (7.1%), and non‐web‐based methods (1.3%). Overall, 450 (25.5%) participants reported no exposure to financial fraud (Group A), 475 (26.9%) reported exposure without monetary loss (Group B), and 839 (47.6%) reported being scammed out of money (Group C).

**TABLE 3:** Types of financial fraud exposure and events of monetary loss (*N* = 1764).

	No exposure *n* (%)	Exposure but no money scammed *n* (%)	Exposure and money scammed *n* (%)
“It's Me” fraud	1255 (71.1)	387 (21.9)	122 (6.9)
Kidnapping fraud	1354 (76.8)	311 (17.6)	99 (5.6)
Refund fraud	1201 (68.1)	398 (22.6)	165 (9.3)
Bank or cell phone account fraud	1132 (64.2)	384 (26.3)	168 (9.5)
Billing fraud	1168 (66.2)	346 (19.6)	250 (14.2)
Shopping fraud	1251 (70.9)	233 (13.2)	280 (15.9)
Financial investment fraud	1247 (70.7)	416 (23.6)	101 (5.7)
Lottery fraud	1269 (71.9)	339 (19.2)	156 (8.8)
Job hunting fraud	1462 (82.9)	210 (11.9)	92 (5.2)
Romance fraud	1447 (82.0)	249 (14.1)	68 (3.9)
Fortune‐telling fraud	1546 (87.6)	146 (8.3)	72 (4.1)

Table 4 presents the results of ANOVA comparing the demographic characteristics and the rate of a self‐reported ADHD diagnosis among the groups with varying experiences of financial fraud victimization. The participants in Group A were more likely to have an education level of senior high school or below than were those in Groups B and C (*p* < 0.001). Participants in Group C were more likely to have a self‐reported ADHD diagnosis than were those in Groups A and B (*p* < 0.001). No significant differences in sex, age, or sexual orientation were noted among the three groups.

**TABLE 4:** Comparisons of demographic characteristics and self‐reported attention‐deficit/hyperactivity disorder (ADHD) among three groups with different financial fraud victimization experiences.

	Group A (*N* = 450)	Group B (*N* = 475)	Group C (*N* = 839)	*p*
Gender (woman), *n* (%)^a^	244 (54.22)	278 (58.53)	464 (55.30)	0.375
Age (years), mean (SD)^b^	22.21 (1.70)	22.31 (1.87)	22.34 (1.72)	0.428
Education (senior high school and below), *n* (%)^a^	87 (19.33)	56 (11.79)	93 (11.08)	< 00.001
Sexual orientation (heterosexual), *n* (%)^a^	361 (80.22)	394 (82.95)	693 (82.60)	0.484
Self‐reported ADHD, *n* (%)^a^	6 (1.33)	11 (2.32)	44 (5.24)	< 0.001

*Note*: Group A: No exposure to fraud; Group B: Exposure to fraud without monetary loss; Group C: Exposure to fraud with monetary loss.

^a^ Chi‐square test.

^b^ Compared using analysis of variance.

Table 5 presents the results of MANCOVA comparing working memory and inhibition among the three groups (Box's *M* = 36.282, *F*(15, 492566.926) = 2.402, *p* = 0.002; Pillai's trace = 0.093, *F*(4, 3520) = 42.979, *p* < 0.001, partial *η* ^2^ = 0.047). Given the significant difference in education levels among the three groups, all multivariate analyses were adjusted for education level, which was entered as a categorical factor (high school or below vs. college or above). After adjustment for education level, the participants in Group C exhibited worse working memory and inhibition than did those in Groups A and B (working memory, Group A vs. C, *p* < 0.001 and Group B vs. C, *p* < 0.001; inhibition, Group A vs. C, *p* < 0.001 and Group B vs. C, *p* < 0.001). Participants in Group B had worse working memory (*p* < 0.001) and inhibition (*p* = 0.016) than did those in Group A.

**TABLE 5:** Comparisons of working memory and inhibition deficits among three groups with different financial fraud victimization experiences^a^.

	Mean (SD)	Partial *η* ^2^	Cohen's *d*	*p* ^b^	Post hoc
Working memory Group A Group B Group C	20.33 (6.29) 22.27 (5.80) 24.85 (6.44)	0.090	0.30^c^ 0.63^c^	< 0.001	Group C > Group B > Group A
Inhibition Group A Group B Group C	12.74 (3.29) 13.23 (3.36) 14.54 (3.34)	0.056	0.09^c^ 0.27^c^	< 0.001	Group C > Group B > Group A

*Note*: Group A: No exposure to fraud; Group B: Exposure to fraud without monetary loss; Group C: Exposure to fraud with monetary loss.

Partial *η* ^2^: > 0.01 = small effect; > 0.06 = medium effect; > 0.14 = large effect.

Cohen's *d*: > 0.2 = small effect; > 0.5 = medium effect; > 0.8 = large effect.

^a^ Compared using multivariate analysis of covariance and adjusted for education level.

^b^ *p* value for test of between groups on each domain of executive function.

^c^ Group A as reference.

Table 6 presents the results of MANCOVA comparing personality traits, assessed using the BFI, among the three groups (Box's *M* = 268.381, *F*(75, 270905.256) = 3.518, *p* < 0.001; Pillai's trace = 0.054, *F*(10, 3514) = 9.689, *p* < 0.001, partial *η* ^2^ = 0.027). After adjustment for education level, the participants in Group A scored higher in terms of extraversion, agreeableness, conscientiousness, and openness than did those in Group C (extraversion, *p* = 0.010; agreeableness, *p* < 0.001; conscientiousness, *p* < 0.001; openness, *p* = 0.023). The participants in Group C scored higher in terms of neuroticism than did those in Group A (*p* = 0.009). In addition, the participants in Group B scored higher in terms of conscientiousness than did those in Group C (*p* = 0.001).

**TABLE 6:** Comparisons of personality traits among three groups with different financial fraud victimization experiences^a^.

	Mean (SD)	Partial *η* ^2^	Cohen's *d*	*p* ^b^	Post hoc
Extraversion Group A Group B Group C	10.23 (2.69) 9.93 (2.22) 9.79 (2.60)	0.005	−0.11^c^ −0.15^c^	0.009	Group A > Group C
Agreeableness Group A Group B Group C	10.77 (2.58) 9.79 (2.35) 10.11 (2.37)	0.023	−0.36^c^ −0.23^c^	< 0.001	Group A > Groups B and C
Conscientiousness Group A Group B Group C	11.76 (2.23) 11.35 (2.06) 10.92 (2.06)	0.027	−0.18^c^ −0.33^c^	< 0.001	Group A > Group B > Group C
Neuroticism Group A Group B Group C	8.56 (2.95) 8.90 (2.61) 9.03 (2.64)	0.005	0.11^c^ 0.14^c^	0.012	Group C > Group A
Openness Group A Group B Group C	9.76 (2.43) 9.44 (2.24) 9.39 (2.51)	0.005	−0.12^c^ −0.13^c^	0.018	Group A > Groups B and Group C

*Note*: Group A: No exposure to fraud; Group B: Exposure to fraud without monetary loss; Group C: Exposure to fraud with monetary loss.

Partial *η* ^2^: > 0.01 = small effect; > 0.06 = medium effect; > 0.14 = large effect.

Cohen's *d*: > 0.2 = small effect; > 0.5 = medium effect; > 0.8 = large effect.

^a^ Compared using multivariate analysis of covariance and adjusted for education level.

^b^ *p* value for test of between groups on each domain of personality traits.

^c^ Group A as reference.

## 4 Discussion

A high proportion of young adults in the present study (47.6%) reported being scammed out of money because of financial fraud. Differences were observed in the executive function, personality traits, and the rate of a self‐reported ADHD diagnosis among the groups with different experiences of financial fraud victimization. Participants who had been scammed were more likely to have a self‐reported ADHD diagnosis and had worse working memory and inhibition than did those in the other groups. Participants who had no exposure to fraud scored higher in extraversion, agreeableness, conscientiousness, and openness than did those who had been scammed. By contrast, participants who had been scammed scored higher in neuroticism than did those with no exposure.

The present study determined that 26.9% of participants reported exposure to financial fraud but without monetary loss and 47.6% reported being scammed out of money in the past 5 years. A previous study demonstrated that 66% of people in Taiwan encounter at least one attempt at fraud every month (Global Anti‐Fraud Alliance 2024). The rate of exposure to financial fraud in this study was compatible with that of the previous survey. Although older adults are traditionally considered to be more vulnerable to financial fraud, the high rate of victimization among the young adults in this study indicates that fraud prevention efforts should not solely focus on the older population; they should also include younger populations. Approximately one‐quarter of this study's participants reported no exposure to financial fraud, whereas another quarter had been exposed but did not lose money. Understanding the factors associated with these differing experiences can help with identifying risk indicators and can inform the development of targeted prevention programs for young adults.

In this study, the participants who had been scammed out of money through financial fraud had worse working memory and inhibition than did those in the other groups. In addition, the participants who had been exposed to fraud but were not scammed had worse working memory than did those with no exposure. Working memory temporarily stores and processes information to support complex cognitive tasks such as reasoning, decision‐making, and learning (Baddeley 1992; Diamond 2013). However, short‐term memory only temporarily stores information, and working memory is able to manipulate and process stored information (Baddeley 1992; Diamond 2013). It is possible that working memory enables an individual to determine whether a message or invitation is potentially fraudulent by enabling them to process the message or invitation on the basis of their personal experiences or prior knowledge. It is also possible that working memory can help an individual detect inconsistencies in fraudulent messages, which can reduce the likelihood of being defrauded. In addition, inhibitory control may help individuals regulate impulses and avoid making poor decisions in the context of potential fraud (Baddeley 1992; Diamond 2013). Particularly in the digital realm, messages relating to financial fraud are often disguised in such a way that recipients find it difficult to distinguish them; working memory and inhibition are especially needed to help a person effectively distinguish between truth and falsehood, recognize scams, and resist being deceived.

The current study revealed that the personality traits of extraversion, agreeableness, conscientiousness, neuroticism, and openness to experience, as measured using the BFI, differed between young adults who had no exposure to fraud and those who had been scammed out of money. Studies have reported that conscientiousness and agreeableness are associated with a lower risk of fraud victimization in older adults (Judges et al. 2017; Xing et al. 2020). Furthermore, this association might be bidirectional. Individuals with high levels of conscientiousness tend to be organized and detail‐oriented (John et al. 1991; R.‐H. Li and Chung 2020); these traits may help them calmly analyze fraudulent messages to determine the messages’ plausibility. In addition, those with high levels of openness are curious and willing to try new things (John et al. 1991; R.‐H. Li and Chung 2020); their exposure to diverse information in the community may enhance their awareness of and alertness to financial fraud. Individuals with high levels of extraversion are more likely to engage in social interactions (John et al. 1991; R.‐H. Li and Chung 2020), which may enable them to learn from others’ experiences and reduce their exposure to fraud. People with high levels of agreeableness are compassionate, cooperative, and empathetic (John et al. 1991; R.‐H. Li and Chung 2020); they may maintain supportive social networks and seek advice when uncertain about the legitimacy of a message. In summary, these protective personality traits may help a person to analyze information calmly, recognize the potential for fraud, engage in appropriate social interactions, and learn new information, thereby reducing the risk of falling victim to financial fraud.

Individuals with high levels of neuroticism have a high tendency to experience negative emotions such as anxiety, sadness, and irritability (John et al. 1991; R.‐H. Li and Chung 2020), which may impair their judgment and increase their vulnerability to fraud. Alternatively, being victimized by financial fraud may negatively affect emotional well‐being and increase neurotic tendencies. Prolonged exposure to financial fraud may also cause victims to perceive ordinary situations as threats and become more sensitive to stress, thereby exacerbating neuroticism. Additional longitudinal studies are required to clarify the temporal association between personality traits and financial fraud exposure.

Previous research has demonstrated that adults with a clinical diagnosis of ADHD experience greater difficulties with various aspects of financial capability (Bangma et al. 2021; Koerts et al. 2023); this may be linked to the core symptoms of ADHD. Inattention, impulsivity, intolerance to delayed rewards, and risky decision‐making are common characteristics among adults with ADHD (Kooij et al. 2010; Mowinckel et al. 2015). Inattention can prevent young adults from noticing suspicious elements of fraudulent messages. Impulsivity and intolerance to delayed rewards may lead them to react quickly, without carefully considering a situation or their actions. Risky decision‐making may result in choices being made without comprehensive evaluation of potential negative consequences. Collectively, it is possible that these ADHD traits increase the likelihood of financial fraud victimization. Further longitudinal studies are needed to clarify the temporal relationship. The diagnosis of ADHD was self‐reported by the participants in this study. A meta‐analysis on 103 published studies found that the overall prevalence of ADHD in register studies was 1.6%, 5.0% in survey studies, 4.2% in one‐stage clinical studies, and 4.8% in two‐stage clinical studies (Popit et al. 2024). By comparison, the proportion (3.5%) of participants in this study who self‐reported having a diagnosis of ADHD might be an underestimate. This study also did not assess the types and severity of the participants’ ADHD symptoms, making it difficult to determine the association between types and severity of ADHD symptoms and the experiences of financial fraud victimization.

### 4.1 Implications

Financial fraud victimization is prevalent among young adults. Given the financial losses and psychological consequences associated with such victimization, prevention strategies such as digital literacy training, cognitive awareness training, and risk perception training must be designed to reduce the risk of financial fraud victimization in the young adult population. Several domains of executive functioning, personality traits, and ADHD are associated with financial fraud victimization and should be considered in the development of prevention programs. Individuals with poor working memory and inhibition, neuroticism, and ADHD may be at risk of financial fraud victimization; prevention programs should help them become more aware of financial fraud and better equipped to deal with it should they fall victim to it. When healthcare professionals encounter individuals with poor working memory and inhibition, neuroticism, and ADHD, they should remind them to be aware of the possibility of fraud. Public awareness campaigns should also alert the public to the risk of fraud faced by people in their lives who exhibit the aforementioned risk factors and provide them with assistance as needed. Additional longitudinal studies are required to examine the temporal relationships between executive functioning, personality traits, ADHD, and financial fraud victimization in young adults.

### 4.2 Strengths and Limitations

There are limited studies in the literature examining the associations of executive functioning, personality traits, and self‐reported ADHD with financial fraud victimization in young adults. However, this study has several limitations that should be noted. First, participant recruitment was conducted through online advertisements, primarily through Dcard, a platform widely used by young adults in Taiwan. Although Dcard has broad reach, its user base may not be fully representative of the general young adult population in Taiwan. For example, in this study, 86.6% of the participants had a college‐level education or higher, indicating that the generalizability of the current findings may be limited. Recruiting participants solely through an online platform and on a voluntary basis increases the risk of selection bias. Second, all data were self‐reported by the participants, introducing the possibility of single‐rater and recall bias, which may have affected the accuracy and reliability of responses. Self‐reported assessment of financial fraud exposure over the past 5 years might introduce recall bias. Third, to prevent respondents from abandoning the online survey midway due to fatigue, this study did not use a questionnaire to screen for ADHD but instead relied on self‐reported ADHD diagnoses. However, self‐reported ADHD by the participants might have been underestimated. The absence of clinical validation or a standardized assessment tool constituted a methodological limitation. Fourth, the cross‐sectional design of this study precludes definitive conclusions from being drawn regarding the temporal associations between executive functioning, personality traits, and financial fraud victimization. Causal relationships between these factors could not be inferred. Longitudinal studies are required to establish causal relationships. Fifth, several potential confounding variables were not controlled in this study. Variables such as socioeconomic status, financial literacy, internet usage intensity, psychiatric comorbidities, and previous exposure to fraud awareness campaigns that may be associated with financial fraud risk should be considered in further studies. Studies have found that financial assets and financial literacy were positively associated with fraud exposure and victimization in Chinese middle‐aged and older people (Mao and Liu 2023; Yu et al. 2023). Heavy internet use may increase the risk of exposure to online financial fraud and becoming a victim. Impaired cognitive function—particularly impaired ability to make financial decisions—is a risk factor for falling victim to fraud in older people (DeLiema 2018; Judges et al. 2017). The Taiwanese government has promoted a three‐stage public education strategy from fraud awareness to fraud prevention to reduce the financial fraud victimization (Chang 2026). However, the effect of fraud awareness campaigns on the prevention of financial fraud victimization warrants examination.

## 5 Conclusion

This study demonstrated that a high proportion of the participating young adults experienced financial fraud victimization. This study also found associations of working memory and inhibition, personality trait, and self‐reported ADHD with financial fraud victimization. Prevention programs should help individuals with poor working memory and inhibition, neuroticism, and ADHD increase the awareness of financial fraud and the ability to deal with fraud.

## Author Contributions

**Yi‐Chun Yeh**: writing – original draft. **Pai‐Cheng Lin**: conceptualization, project administration. **Chung‐Ying Lin**: formal analysis. **Yu‐Ping Chang**: writing – review and editing. **Peng‐Wei Wang**: formal analysis, writing – original draft. **Cheng‐Fang Yen**: data curation, methodology.

## Funding

This study was supported by a grant from Kaohsiung Medical University Gangshan Hospital (KMUGH‐114‐043).

## Conflicts of Interest

The authors declare no conflicts of interest.

## Supporting information




**Supplementary Tables**: brb371780‐sup‐0001‐SuppMat.docx

## Data Availability

Data will be made available on request.

## References

[brb371780-bib-0001] Academy for the Judiciary, Ministry of Justice . 2023. The Analysis of Internet Fraud Victimization and Preventive Strategies in Taiwan. http://chrome‐extension/efaidnbmnnnibpcajpcglclefindmkaj/. https://www.cprc.moj.gov.tw/media/20213323/112%E5%B9%B4%E5%A7%94%E8%A8%97%E7%A0%94%E7%A9%B6%E6%A1%88‐%E8%B3%B4%E6%93%81%E9%80%A3%E7%AD%89‐%E6%88%91%E5%9C%8B%E7%B6%B2%E8%B7%AF%E8%A9%90%E6%AC%BA%E8%A2%AB%E5%AE%B3%E8%AA%BF%E6%9F%A5%E8%88%87%E9%98%B2%E5%88%B6%E7%A0%94%E7%A9%B6.pdf?mediaDL=true.

[brb371780-bib-0002] Baddeley, A. 1992. “Working Memory.” Science 255, no. 5044: 556–559. 10.1126/science.1736359. 1736359

[brb371780-bib-0003] Bangma, D. F. , O. Tucha , L. Tucha , P. P. De Deyn , and J. Koerts . 2021. “How Well Do People Living With Neurodegenerative Diseases Manage Their Finances? A Meta‐Analysis and Systematic Review on the Capacity to Make Financial Decisions in People Living With Neurodegenerative Diseases.” Neuroscience & Biobehavioral Reviews 127: 709–739. 10.1016/j.neubiorev.2021.05.021. 34058557

[brb371780-bib-0004] Bar Lev, E. , L.‐G. Maha , and S.‐C. Topliceanu . 2022. “Financial Frauds' Victim Profiles in Developing Countries.” Frontiers in Psychology 13: 999053. 10.3389/fpsyg.2022.999053 PMC9592820 36304867

[brb371780-bib-0005] Boyle, P. A. , L. Yu , R. S. Wilson , K. Gamble , A. S. Buchman , and D. A. Bennett . 2012. “Poor Decision Making is a Consequence of Cognitive Decline Among Older Persons Without Alzheimer's Disease or Mild Cognitive Impairment.” PLoS ONE 7, no. 8: e43647. 10.1371/journal.pone.0043647. PMC3423371 22916287

[brb371780-bib-0006] Brenner, L. , T. Meyll , O. Stolper , and A. Walter . 2020. “Consumer Fraud Victimization and Financial Well‐Being.” Journal of Economic Psychology 76: 102243. 10.1016/j.joep.2019.102243.

[brb371780-bib-0007] Burnes, D. , C. R. Henderson Jr. , C. Sheppard , R. Zhao , K. Pillemer , and M. S. Lachs . 2017. “Prevalence of Financial Fraud and Scams Among Older Adults in the United States: a Systematic Review and Meta‐analysis.” American journal of public health 107, no. 8: e13–e21. 10.2105/AJPH.2017.303821. PMC5508139 28640686

[brb371780-bib-0008] Button, M. , C. Lewis , and J. Tapley . 2014. “Not a Victimless Crime: The Impact of Fraud on Individual Victims and Their Families.” Security Journal 27, no. 1: 36–54. 10.1057/sj.2012.11.

[brb371780-bib-0009] Calso, C. , J. Besnard , and P. Allain . 2020. “Study of the Theory of Mind in Normal Aging: Focus on the Deception Detection and its Links With Other Cognitive Functions.” Aging, Neuropsychology, and Cognition 27, no. 3: 430–452. 10.1080/13825585.2019.1628176. 31188065

[brb371780-bib-0010] Chang, M.‐H. 2026. “Safeguarding Capital Market Resilience: TWSE's “5D Anti‐Fraud Campaign” Builds a Public Safety Net Against Investment Scams.” Taiwan Stock Exchange Corporation. https://www.twse.com.tw/market_insights/en/detail/8a8216d69e6379f4019e6cbbcf4a005c.

[brb371780-bib-0011] Cross, C. 2015. “No Laughing Matter: Blaming the Victim of Online Fraud.” International Review of Victimology 21, no. 2: 187–204. 10.1177/0269758015571471.

[brb371780-bib-0012] DeLiema, M. 2018. “Elder Fraud and Financial Exploitation: Application of Routine Activity Theory.” The Gerontologist 58, no. 4: 706–718. 10.1093/geront/gnw258. PMC6044329 28329818

[brb371780-bib-0013] DeLiema, M. , M. Deevy , A. Lusardi , and O. S. Mitchell . 2017. “Exploring the Risks and Consequences of Elder Fraud Victimization: Evidence From the Health and Retirement Study.” Working Paper no. 2018‐06 and Research Paper No. 2017‐364. Michigan Retirement Research Center, Wharton Pension Research Council, December 1.

[brb371780-bib-0014] DeLiema, M. , L. Langton , D. Brannock , and E. Preble . 2024. “Fraud Victimization Across the Lifespan: Evidence on Repeat Victimization Using Perpetrator Data.” Journal of Elder Abuse & Neglect 36, no. 3: 227–250. 10.1080/08946566.2024.2321923. 38389208

[brb371780-bib-0015] Diamond, A. 2013. “Executive Functions.” Annual Review of Psychology 64: 135–168. 10.1146/annurev-psych-113011-143750. PMC4084861 23020641

[brb371780-bib-0016] Ebner, N. C. , D. M. Ellis , T. Lin , et al. 2020. “Uncovering Susceptibility Risk to Online Deception in Aging.” The Journals of Gerontology: Series B 75, no. 3: 522–533. 10.1093/geronb/gby036. PMC8921760 29669133

[brb371780-bib-0017] Fan, J. X. , and Z. Yu . 2021. “Understanding Aging and Consumer Fraud Victimization in the Chinese Context: A Two‐Stage Conceptual Approach.” Journal of Elder Abuse & Neglect 33, no. 3: 230–247. 10.1080/08946566.2021.1937428. 34148521

[brb371780-bib-0018] Global Anti‐Fraud Alliance . 2024. “Asia Scam Report—2024.” https://gasa.org/knowledge‐base/reports/asia‐scam‐report‐2024.

[brb371780-bib-0019] Hall, J. , D. L. Karch , and A. Crosby . 2016. Elder Abuse Surveillance: Uniform Definitions and Recommended Core Data Elements for use in Elder Abuse Surveillance, Version 1.0. CDC.

[brb371780-bib-0020] Hamby, S. , Z. Blount , E. Taylor , K. Mitchell , and L. Jones . 2021. “The Association of Different Cyber‐Victimization Types With Current Psychological and Health Status in Southern Appalachian Communities.” Violence and Victims 36, no. 2: 251–271. 10.1891/vv-d-18-00214. 33361446

[brb371780-bib-0021] Han, S. D. , P. A. Boyle , B. D. James , L. Yu , and D. A. Bennett . 2015a. “Mild Cognitive Impairment is Associated With Poorer Decision‐Making in Community‐Based Older Persons.” Journal of the American Geriatrics Society 63, no. 4: 676–683. 10.1111/jgs.13346. PMC4406791 25850350

[brb371780-bib-0022] Han, S. D. , P. A. Boyle , B. D. James , L. Yu , and D. A. Bennett . 2015b. “Poorer Financial and Health Literacy among Community‐Dwelling Older Adults With Mild Cognitive Impairment.” Journal of Aging and Health 27, no. 6: 1105–1117. 10.1177/0898264315577780. PMC4520748 25903976

[brb371780-bib-0023] Han, S. D. , P. A. Boyle , B. D. James , L. Yu , and D. A. Bennett . 2016. “Mild Cognitive Impairment and Susceptibility to Scams in Old Age.” Journal of Alzheimer's Disease 49, no. 3: 845–851. 10.3233/jad-150442. PMC4708889 26519434

[brb371780-bib-0024] Hanoch, Y. , and S. Wood . 2021. “The Scams Among Us: Who Falls Prey and Why.” Current Directions in Psychological Science 30, no. 3: 260–266. 10.1177/0963721421995489.

[brb371780-bib-0025] Holst, Y. , and L. B. Thorell . 2018. “Adult Executive Functioning Inventory (ADEXI): Validity, Reliability, and Relations to ADHD.” International Journal of Methods in Psychiatric Research 27, no. 1: e1567. 10.1002/mpr.1567. PMC6877129 28497641

[brb371780-bib-0026] John, O. P. , E. M. Donahue , and R. L. Kentle . 1991. The Big‐Five Inventory‐Version 4a and 54. Berkeley, CA: Berkeley Institute of Personality and Social Research, University of California.

[brb371780-bib-0027] Judges, R. A. , S. N. Gallant , L. Yang , and K. Lee . 2017. “The Role of Cognition, Personality, and Trust in Fraud Victimization in Older Adults.” Frontiers in Psychology 8: 588. 10.3389/fpsyg.2017.00588. PMC5390488 28450847

[brb371780-bib-0028] Kemp, S. , and N. Erades Pérez . 2023. “Consumer Fraud Against Older Adults in Digital Society: Examining Victimization and Its Impact.” International Journal of Environmental Research and Public Health 20, no. 7: 5404. 10.3390/ijerph20075404. PMC10094555 37048017

[brb371780-bib-0029] Kim, H. Y. 2013. “Statistical Notes for Clinical Researchers: Assessing Normal Distribution (2) Using Skewness and Kurtosis.” Restorative Dentistry & Endodontics 38, no. 1: 52. 10.5395/rde.2013.38.1.52. PMC3591587 23495371

[brb371780-bib-0030] Knupfer, S. , V. Rantala , and P. Vokata . 2021. Scammed and Scarred: Effects of Investment Fraud on Its Victims. Ohio State University, Charles A. Dice Center for Research in Financial Economics.

[brb371780-bib-0031] Koerts, J. , D. F. Bangma , C. Mette , L. Tucha , and O. Tucha . 2023. “Strengths and Weaknesses of Everyday Financial Knowledge and Judgment Skills of Adults With ADHD.” International Journal of Environmental Research and Public Health 20, no. 5: 4656. 10.3390/ijerph20054656. PMC10001631 36901665

[brb371780-bib-0032] Kooij, S. J. , S. Bejerot , A. Blackwell , et al. 2010. “European Consensus Statement on Diagnosis and Treatment of Adult ADHD: The European Network Adult ADHD.” BMC Psychiatry 10: 67. 10.1186/1471-244x-10-67. PMC2942810 20815868

[brb371780-bib-0033] Li, J. C. M. , G. T. W. Wong , M. Manning , and D. Y. Yeung . 2022. “Financial Fraud Against Older People in Hong Kong: Assessing and Predicting the Fear and Perceived Risk of Victimization.” International Journal of Environmental Research and Public Health 19, no. 3: 1233. 10.3390/ijerph19031233. PMC8835379 35162252

[brb371780-bib-0034] Li, R.‐H. , and H.‐Y. Chung . 2020. “The Development of a Chinese Shortened Version of the Big Five Inventory (BFI).” Psychological Testing 67, no. 4: 271–299.

[brb371780-bib-0035] Mao, X. , and Y. Liu . 2023. “Relationship Between Socioeconomic Conditions and Financial Fraud Victimization Among Older Adults in China: Do Financial Literacy and Financial Attitudes Matter?” Research on Aging 45, no. 7–8: 503–516. 10.1177/01640275221132195. 36198441

[brb371780-bib-0036] Mowinckel, A. M. , M. L. Pedersen , E. Eilertsen , and G. Biele . 2015. “A Meta‐Analysis of Decision‐Making and Attention in Adults With ADHD.” Journal of Attention Disorders 19, no. 5: 355–367. 10.1177/1087054714558872. 25477020

[brb371780-bib-0037] National Police Agency, Ministry of the Interior . 2025. Anti‐Fraud 165 Dashboard. https://165dashboard.tw/.

[brb371780-bib-0038] Popit, S. , K. Serod , I. Locatelli , and M. Stuhec . 2024. “Prevalence of Attention‐Deficit Hyperactivity Disorder (ADHD): Systematic Review and Meta‐Analysis.” European Psychiatry 67, no. 1: e68. 10.1192/j.eurpsy.2024.1786. PMC11536208 39381949

[brb371780-bib-0039] Tabachnick, B. G. , and L. S. Fidell . 2018. Using Multivariate Statistics. Pearson.

[brb371780-bib-0040] Townes, P. , C. Liu , P. Panesar , et al. 2023. “Do ASD and ADHD Have Distinct Executive Function Deficits? A Systematic Review and Meta‐Analysis of Direct Comparison Studies.” Journal of Attention Disorders 27, no. 14: 1571–1582. 10.1177/10870547231190494. PMC10637091 37565325

[brb371780-bib-0041] Ueno, D. , Y. Daiku , Y. Eguchi , et al. 2021. “Mild Cognitive Decline is a Risk Factor for Scam Vulnerability in Older Adults.” Frontiers in Psychiatry 12: 685451. 10.3389/fpsyt.2021.685451. PMC8721598 34987422

[brb371780-bib-0042] Wan Nor, A. 2015. “The Graphical Assessment of Multivariate Normality Using SPSS.” Education in Medicine Journal 7, no. 2: e71–e75. 10.5959/eimj.v7i2.361.

[brb371780-bib-0043] Whitty, M. T. 2019. “Predicting Susceptibility to Cyber‐Fraud Victimhood.” Journal of Financial Crime 26, no. 1: 277–292. 10.1108/JFC-10-2017-0095.

[brb371780-bib-0044] Xing, T. , F. Sun , K. Wang , J. Zhao , M. Wu , and J. Wu . 2020. “Vulnerability to Fraud Among Chinese Older Adults: Do Personality Traits and Loneliness Matter?” Journal of Elder Abuse & Neglect 32, no. 1: 46–59. 10.1080/08946566.2020.1731042. 32079509

[brb371780-bib-0045] Yu, S. J. , C. T. Kuo , and Y. C. Tseng . 2023. “Association of Financial Literacy and Risk Preference With Fraud Exposure and Victimization Among Middle‐Aged and Older Adults in China.” Journal of Applied Gerontology 42, no. 1: 89–98. 10.1177/07334648221121775. 36007017

