# Supplementary material for: Financial Fraud Victimization Among Young Adults in Taiwan: Roles of Executive Functioning, Personality Traits, and the Self‐Reported Attention‐Deficit/Hyperactivity Disorder Diagnosis
Source: Brain Behav. 2026 Sep 23;16(9):e71780. doi: 10.1002/brb3.71780 (PMC13601758; doi:10.1002/brb3.71780)
Supplement: Supplementary file 1 [file BRB3-16-e71780-s001.docx]

**Supplementary Table 1**

*Fit Statistics in Confirmatory Factor Analysis and Internal Consistency*

| Statistics | Values from the present sample | Acceptable cutoff |
| --- | --- | --- |
| χ^2^ (*df*), *p* value | 95.637 (44), *p* < .001 | Nonsignificant |
| CFI | 0.951 | 0.9 |
| TLI | 0.938 | 0.9 |
| RMSEA (90% CI) | 0.036 (0.026, 0.045) | 0.08 |
| SRMR | 0.053 | 0.08 |
| Cronbach’s α | 0.847 | 0.7 |
| McDonald’s ω | 0.878 | 0.7 |

Note. CFI = comparative fit index; TLI = Tucker-Lewis index; RMSEA = root mean square error of approximation; SRMR = standardized root mean square residual; CI = confidence interval.

| **Supplementary Table 2**  *Sensitivity Analyses Using Heteroskedasticity-Consistent Standard Errors* | | | | | | |
| --- | --- | --- | --- | --- | --- | --- |
|  |  | B^a^ | SE^b^ | t | *p* | 95% CI |
| Working memory |  |  |  |  |  |  |
|  | Group C vs Group A | 4.68 | .37 | 12.553 | < .001 | 3.95 to 5.41 |
|  | Group B vs Group A | 2.09 | .40 | 5.226 | < .001 | 1.30 to 2.87 |
| Inhibition |  |  |  |  |  |  |
|  | Group C vs Group A | 1.85 | .20 | 9.481 | < .001 | 1.46 to 2.23 |
|  | Group B vs Group A | .53 | .22 | 2.422 | .016 | .10 to 96 |
| Extraversion |  |  |  |  |  |  |
|  | Group C vs Group A | -.46 | .16 | -2.900 | .004 | -.76 to -.15 |
|  | Group B vs Group A | -.32 | .16 | -1.957 | .051 | -.64 to .001 |
| Agreeableness |  |  |  |  |  |  |
|  | Group C vs Group A | -.67 | .15 | -4.545 | < .001 | -.97 to -.38 |
|  | Group B vs Group A | -1.00 | .16 | -6.101 | < .001 | -1.32 to -.68 |
| Conscientiousness |  |  |  |  |  |  |
|  | Group C vs Group A | -.85 | .13 | -6.659 | < .001 | -1.10 to -.60 |
|  | Group B vs Group A | -.42 | .14 | -2.994 | .003 | -.70 to -.15 |
| Neuroticism |  |  |  |  |  |  |
|  | Group C vs Group A | .47 | .17 | 2.850 | .004 | .15 to .80 |
|  | Group B vs Group A | .35 | .19 | 1.888 | 0.59 | -.01 to .71 |
| Openness |  |  |  |  |  |  |
|  | Group C vs Group A | -.39 | .15 | -2.707 | .007 | -.68 to -.11 |
|  | Group B vs Group A | -.34 | .15 | -2.169 | .030 | -.64 to -.03 |
| a: adjusted mean difference; b: HC3 method | | | | | | |
